# Research on Deep Reinforcement Learning Control Algorithm for Active Suspension Considering Uncertain Time Delay

**DOI:** 10.3390/s23187827

**Published:** 2023-09-12

**Authors:** Yang Wang, Cheng Wang, Shijie Zhao, Konghui Guo

**Affiliations:** 1 School of Mechanical Engineering, Beijing Institute of Technology, Beijing 100811, China; 2 State Key Laboratory of Automotive Simulation and Control, Jilin University, Changchun 130025, China

**Keywords:** active suspension, deep reinforcement learning, suspension control, uncertain time delay

## Abstract

The uncertain delay characteristic of actuators is a critical factor that affects the control effectiveness of the active suspension system. Therefore, it is crucial to develop a control algorithm that takes into account this uncertain delay in order to ensure stable control performance. This study presents a novel active suspension control algorithm based on deep reinforcement learning (DRL) that specifically addresses the issue of uncertain delay. In this approach, a twin-delayed deep deterministic policy gradient (TD3) algorithm with system delay is employed to obtain the optimal control policy by iteratively solving the dynamic model of the active suspension system, considering the delay. Furthermore, three different operating conditions were designed for simulation to evaluate the control performance: deterministic delay, semi-regular delay, and uncertain delay. The experimental results demonstrate that the proposed algorithm achieves excellent control performance under various operating conditions. Compared to passive suspension, the optimization of body vertical acceleration is improved by more than 30%, and the proposed algorithm effectively mitigates body vibration in the low frequency range. It consistently maintains a more than 30% improvement in ride comfort optimization even under the most severe operating conditions and at different speeds, demonstrating the algorithm’s potential for practical application.

## 1. Introduction

With the development of microprocessor, sensor, and actuator technologies, scholars have studied various aspects of active suspension systems. The active suspension system can adaptively adjust the output force by controlling the actuator according to the driving operation and road conditions, achieving both ride comfort and better vehicle driving performance. The active suspension system control has significant prospects due to its ability to meet the comfort and safety requirements of modern vehicles.

Due to the closed-loop nature of the active suspension system, which includes sensors, controllers, and actuators, there are inherent delays in the measured signal or actuator. In the majority of cases, the time delay can be disregarded as it is insignificant. However, there are situations where the magnitude of the time delay is comparable to the control cycle, making it impossible to ignore and requiring careful consideration. Time delays tend to degrade control performance and potentially induce instability in the control system [1,2,3,4]. Therefore, for active suspension systems with delay, developing control strategies that resist uncertain time delays is particularly important.

Currently, delay-sensitive control systems have made significant progress in multiple industries including transportation [5,6], autonomous vehicle control [7], wireless sensor networks [8], and power systems [9,10]. In recent years, stability analysis and controller integration have been used in linear systems with measurement delays or actuator delays. In general, there are two main approaches to dealing with the problem of system delays. One is to design the controller using an integrated system model that includes actuator dynamics [11], and the other is to consider system delays during the controller design process. Ji et al. [12] proposed an improved variable universe fuzzy control strategy with real-time adjustment of the contracting–expanding factor parameters to improve the ride comfort of vehicles. Some scholars have proposed the use of delay compensation techniques to reduce or offset the negative effects of delays [13,14,15]. Udwadia et al. [16] proposed the application of delayed state positive feedback proportional control to the active control of structures. Pan et al. [17] designed a suspension delay active controller using an adaptive control strategy. Some scholars [18,19] have also attempted to design delayed instantaneous optimal control laws for suspension systems with delay using a state transformation approach to ensure the stability of the system. Du et al. [20] designed full-frequency domain state feedback controllers considering input delay for automotive suspensions and seat suspensions and achieved good damping effects within a certain delay range. Li et al. [21], on the other hand, proposed a time-delayed full-frequency domain robust control method based on dynamic output feedback. In addition, Kim et al. [22] combined perception technology and proposed a model predictive control of a semi-active suspension with a shift delay compensation using preview road information. Wu et al. [23] proposed a time-delay control strategy and the idea of using a linear motor as the actuator. According to this idea, they proposed a linear equivalent excitation method to optimize the optimal time-delay control parameters under complex excitation. Li et al. [24] proposed a fuzzy cooperative control strategy based on linear matrix inequality theory to weaken the effect of perturbations on vehicle driving conditions by integrating the problems of variable structure, disturbance immunity, and time delay. Based on Lyapunov theory and backstepping technique, Wang [25] studied the adaptive control problem of nonlinear active suspension systems with random perturbations and time delay. Moreover, some scholars have considered the time-delay characteristics of active suspension systems and controlled them according to the Takagi–Sugeno fuzzy model [26,27,28]. Further, scholars have been searching for more robust control methods to ensure the stability and performance of the suspension system [29,30,31].

Although numerous scholars have conducted extensive research on the problem of delay, the delay control system remains enigmatic due to its infinite-dimensional nature and the inherent uncertainty associated with delay. Therefore, further in-depth research is essential. Simultaneously, the automotive industry is rapidly adopting intelligence as part of its processes, which has led to the integration of artificial intelligence (AI) technology as a solution for controlling complex and uncertain systems. Notably, deep reinforcement learning (DRL) techniques have demonstrated significant advantages in addressing high-dimensional problems. By combining the robust perception and information-processing capabilities of deep learning (DL) with the decision-making proficiency of reinforcement learning (RL) in complex environments, these techniques are gradually gaining traction in the study of various intricate systems.

Currently, DRL has shown its advantages in vision and decision-making [32,33]. Some scholars have also tried to apply it to suspension control [34,35]. In an intuitive application, Pang et al. [36] proposed a non-fragile fault-tolerant control design for Markov-type systems. Providing more depth, Kozek et al. [37] proposed a neural algorithm based on reinforcement learning to optimize the creation of a linear quadratic regulator (LQR). In recent years, Li et al. [38] used an actor–critic architecture to study the adaptive neural network output feedback optimal control problem. In addition, many scholars have utilized different DRL architectures for active or semi-active suspension control. Lin et al. [39] studied a deep deterministic policy gradient (DDPG) control strategy for a full-vehicle active Macpherson suspension system. Yong et al. [40] proposed learning and control strategies for a semi-active suspension system in a full car using soft actor–critic (SAC) models on real roads. Similarly, Lee et al. [41] conducted a study on semi-active suspension control using DRL and proposed a state-normalization filter to improve the generalization performance. Further, Du et al. [42] proposed the utilization of external knowledge in the DDPG framework for suspension control and integration of speed planning to ensure ride comfort. It is worth mentioning that Han et al. [43] and Dridi et al. [44] have tried to use a proximal policy optimization (PPO) algorithm for semi-active and active suspension control and achieved satisfactory control results. Although there have been some useful studies on the application of DRL to suspension control, few attempts have been made to solve the delay problem. However, it is worth noting that in sequential decisions such as DRL control, the delay problem can have a significant impact on the actual control effect. Therefore, using DRL to solve the delay problem is still a new idea to be investigated. In this study, by adding a delay link to the twin-delayed deep deterministic policy gradient (TD3) algorithm, the agent is guided to explore the possibility of obtaining a more robust control strategy in a time-delayed environment, and then effectively suppress the effect of uncertain delay on the active suspension system. To summarize, the main innovations of this study are as follows:

1. To our knowledge, this study represents the first research endeavor to employ DRL techniques within the realm of delay control. The primary aim of this investigation is to alleviate the repercussions of uncertain delays through the implementation of active suspension control strategies rooted in DRL. Furthermore, this study demonstrates the utilization of high-dimensional advantages of DRL in an infinite-dimensional delay control system, ultimately achieving commendable results.
2. In this study, multiple sets of simulation considering deterministic delay, semi-regular delay, and uncertain delay are proposed to test the control performance of the algorithm. Various delay characteristics and uncertainty of the control system under their influence are considered to make the simulation closer to the actual working conditions.
3. The control algorithm proposed in this study maintains good control performance in multiple sets of the simulation built by MATLAB/Simulink for different working conditions and speeds, proving its application potential.

This paper is organized as follows. Section 2 presents the active suspension quarter model and road model. The proposed controller algorithm and the model design associated with it are presented in Section 3 and Section 4, respectively. In Section 5, the simulation environments with deterministic, semi-regular, and uncertain delays set up to validate the performance of the algorithm and their results are shown. In Section 6, the simulation results are discussed in a broader context with respect to the simulation results. Finally, Section 7 provides the conclusions of this study. A table of notations and abbreviations used in this paper is provided in Appendix A.

## 2. Dynamics Model of Active Suspension System Considering Time Delay

### 2.1. Active Suspension Quarter Model

In the field of control algorithms for active suspension systems, the two-degrees-of-freedom quarter suspension has emerged as the established benchmark model for experimentation. This model offers simplicity, and despite only addressing the vertical vibration of the sprung and unsprung masses, it provides an intuitive representation of the algorithm’s impact on control performance. Consequently, it aids researchers in algorithm development. Further, the vertical vibration of the vehicle body is the primary determinant of ride comfort, while the complex structure and pitch-and-roll motion of the body are presently disregarded. Hence, the quarter suspension serves as the foundational model for developing a dynamic model that accounts for delay.

**Figure 1:**
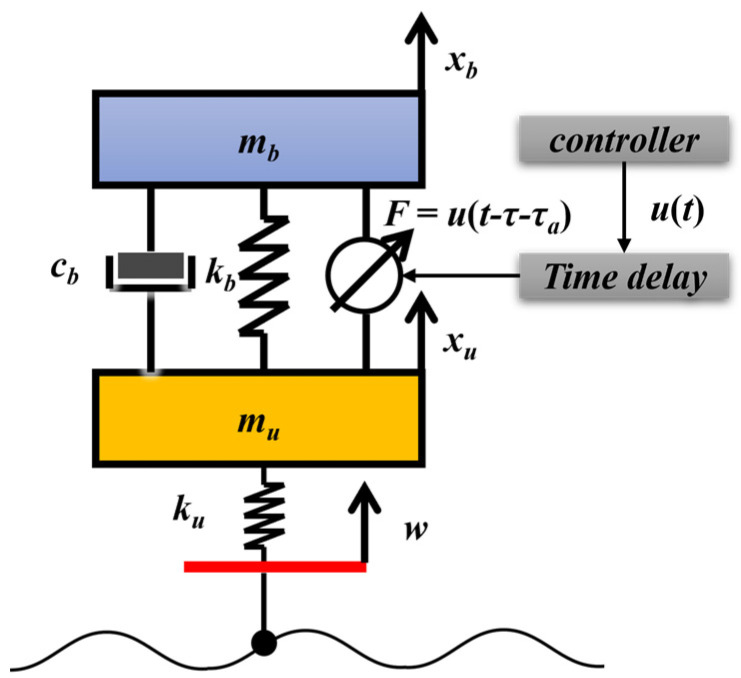
Dynamics model considering delay time.

Typically, models that take into account time delays are generally constructed from Takagi–Sugeno fuzzy models or include a model of an adjustable damper with hysteresis in the dynamics model [45,46,47]. The two-degrees-of-freedom active suspension dynamics model considering delay used in this study is shown in Figure 1. In this model, the system state delay is too small in magnitude, so it can be neglected, and only the inherent delay τ and actuator delay $\tau_{a}$ in the control system are considered. Among them, the inherent delay is the amount of delay caused by the acquisition and transmission of signals in the active suspension control system and the controller operation, and the actuator delay is the amount of delay caused by the actuator response delay, which is unavoidable in the control loop.

According to the second class of Lagrangian equations, the kinetic equation with delayed quantities at moment *t* can be obtained as

(1) $$\{\begin{array}{c} m_{b}{\ddot{x}}_{b}\left(t\right)+k_{b}\left(x_{b}\left(t\right)-x_{u}\left(t\right)\right)+c_{b}\left({\dot{x}}_{b}\left(t\right)-{\dot{x}}_{u}\left(t\right)\right)+u\left(t-\tau -\tau_{a}\right)=0\\ m_{u}{\ddot{x}}_{u}\left(t\right)+k_{b}\left(x_{u}\left(t\right)-x_{b}\left(t\right)\right)+k_{u}\left(x_{u}\left(t\right)-w\left(t\right)\right)+c_{b}\left({\dot{x}}_{u}\left(t\right)-{\dot{x}}_{b}\left(t\right)\right)-u\left(t-\tau -\tau_{a}\right)=0 \end{array}$$

where $m_{b}$ is the sprung mass, kg. $m_{u}$ is the unsprung mass, kg. $k_{b}$ is the spring stiffness, N/m. $k_{u}$ is the equivalent tire stiffness, N/m. $c_{b}$ is the equivalent damping factor of the suspension damping element, N·s/m. $x_{b}$ is the vertical displacement of the sprung mass, m. $x_{u}$ is the vertical displacement of the unsprung mass, m. w is the vertical tire displacement, which can also be equated to the road displacement, m. $F=u\left(t-\tau -\tau_{a}\right)$ is the active suspension actuator control force for the time-delay system, N.

Take the system state variables as

(2) $$x\left(t\right)={\left[\begin{array}{cccc} x_{b}\left(t\right) & x_{u}\left(t\right) & {\dot{x}}_{b}\left(t\right) & {\dot{x}}_{u}\left(t\right) \end{array}\right]}^{T}$$

Take the system output variable as

(3) $$y\left(t\right)={\left[\begin{array}{ccccc} {\ddot{x}}_{b}\left(t\right) & {\dot{x}}_{b}\left(t\right) & {\dot{x}}_{b}\left(t\right)-{\dot{x}}_{u}\left(t\right) & x_{b}\left(t\right)-x_{u}\left(t\right) & x_{u}\left(t\right)-w\left(t\right) \end{array}\right]}^{T}$$

Then, the state space expression of the active suspension, considering the delay, is

(4) $$\{\begin{array}{c} \dot{x}\left(t\right)=Ax\left(t\right)+Bu\left(t-\tau -\tau_{a}\right)+Ew\left(t\right)\\ y\left(t\right)=Cx\left(t\right)+Du\left(t-\tau -\tau_{a}\right)+Lw\left(t\right) \end{array}$$

where $A=\left[\begin{array}{cccc} 0 & 0 & 1 & 0\\ 0 & 0 & 0 & 1\\ -\frac{k_{b}}{m_{b}} & \frac{k_{b}}{m_{b}} & -\frac{c_{b}}{m_{b}} & \frac{c_{b}}{m_{b}}\\ \frac{k_{b}}{m_{u}} & -\frac{\left(k_{b}+k_{u}\right)}{m_{u}} & \frac{c_{b}}{m_{u}} & -\frac{c_{b}}{m_{u}} \end{array}\right]$, $B=\left[\begin{array}{c} 0\\ 0\\ -\frac{1}{m_{b}}\\ \frac{1}{m_{u}} \end{array}\right]$, $E=\left[\begin{array}{c} 0\\ 0\\ 0\\ \frac{k_{u}}{m_{u}} \end{array}\right]$, $C=\left[\begin{array}{cccc} -\frac{k_{b}}{m_{b}} & \frac{k_{b}}{m_{b}} & -\frac{c_{b}}{m_{b}} & \frac{c_{b}}{m_{b}}\\ 0 & 0 & 1 & 0\\ 0 & 0 & 1 & -1\\ 1 & -1 & 0 & 0\\ 0 & 1 & 0 & 0 \end{array}\right]$, $D=\left[\begin{array}{c} -\frac{1}{m_{b}}\\ 0\\ 0\\ 0\\ 0 \end{array}\right]$, $L=\left[\begin{array}{c} 0\\ 0\\ 0\\ 0\\ -1 \end{array}\right]$.

### 2.2. Road Model

The vehicle road model uses a filtered white noise time domain road input model, i.e.,

(5) $$\dot{w}\left(t\right)=-2\pi f_{0}w\left(t\right)+2\pi \sqrt{S_{q}\left(n_{0}\right)v}w_{0}\left(t\right)$$

where w(t) is the road displacement. $f_{0}$ is the lower cutoff frequency. $S_{q}\left(n_{0}\right)$ is the road unevenness coefficient, which is related to the road class, where the unevenness coefficients of class A, B, and C roads are 16, 64, and 256, respectively. v is the speed, m/s. $w_{0}$ is a uniformly distributed white noise with a mean of 0 and an intensity of 1.

The lower cutoff frequency is calculated as

(6) $$f_{0}=2\pi n_{00}v$$

where $n_{00}$ is the spatial cutoff frequency of the pavement, $n_{00}=0.011\;m^{-1}$.

**Figure 2:**
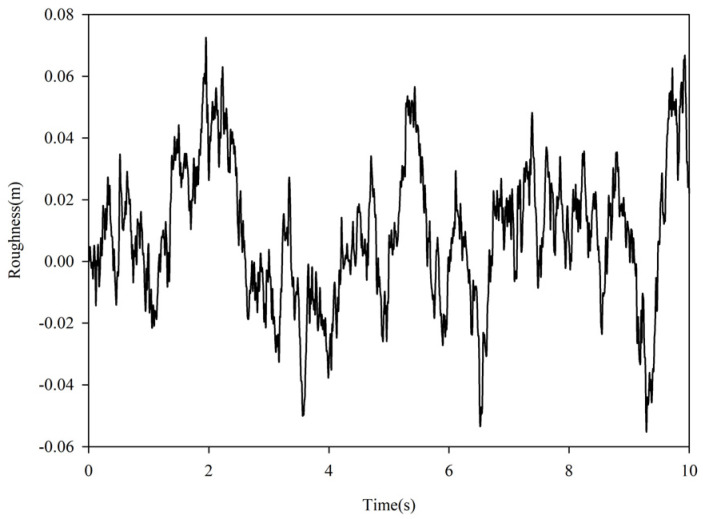
Road time domain unevenness curve.

In this study, the time domain road unevenness curve used by the training agent is shown in Figure 2.

## 3. Controller Algorithm

This section introduces the primary algorithm employed in constructing the controller for the active suspension delay system. Initially, the fundamental principles and framework of reinforcement learning (RL) are presented, followed by an elucidation of the advantages offered by the deep reinforcement learning (DRL) algorithm compared to traditional RL algorithms. Subsequently, the TD3 algorithm, known for its suitability in continuous control systems, is selected based on the characteristics of the delay control system. The algorithmic process and technical intricacies of TD3 are then described.

### 3.1. Reinforcement Learning

**Figure 3:**
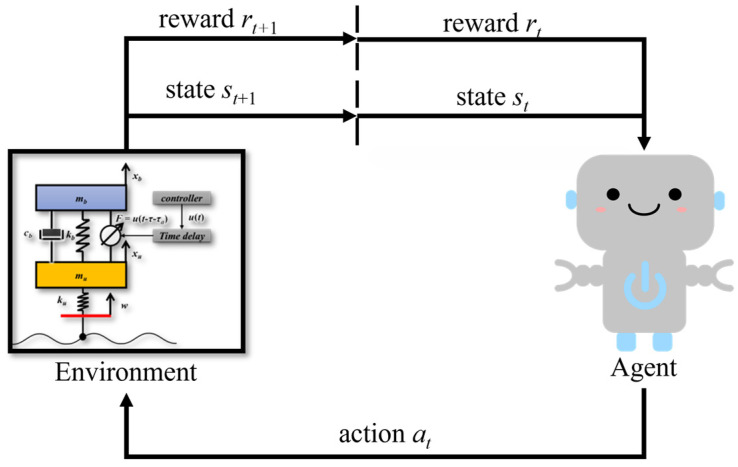
RL basic framework.

RL is a payoff learning method developed from traditional attempted learning, and its basic principles can be traced back to optimal control theory and Markov decision processes (MDP). The RL can be represented as an interactive system consisting of an agent and an environment, as shown in Figure 3. At time t, the environment generates information describing the state of the system, which is the state $s_{t}$. The agent interacts with the environment by observing the state and using this information to select the action $a_{t}$. The environment accepts the action and transitions to the next state $s_{t+1}$. The environment then feeds the next state and its reward $r_{t}$ to the agent. The cycle repeats and iterates continuously until the environment terminates. RL explores learning the optimal policy by maximizing the total reward in the process.

### 3.2. Deep Reinforcement Learning

In RL, the agent learns a function to formulate appropriate actions to maximize the goal. However, RL has limitations when dealing with large-scale problems. For example, in cases where the state space and action space are extremely large, traditional RL algorithms may face the issues of the curse of dimensionality and computational complexity. Additionally, traditional RL algorithms may require a large number of samples and time to learn a good policy, which can be time-consuming and inefficient for certain tasks. With the development of DL technology, we can use deep neural networks as function-approximation methods to learn value functions that imply multidimensional information. It extends the application of RL to higher dimensions, allowing it to suppress the high-dimensional disasters of traditional optimization problems to some extent.

The three primary functions that can be learned in RL align with the three primary methods in DRL, namely, policy-based, value-based, and model-based. Researchers use these three major classes of methods individually or in combination to meet the needs of practical tasks. In this study, the active suspension control system considering delay is a typical continuous state and continuous action control system. The classical DRL algorithm in this field is the DDPG [48]. DDPG utilizes an actor–critic architecture for learning, i.e., an actor network is added to the deep Q network (DQN) [49,50] to output action values directly. It helps the agent to obtain more feedback information and thus make more accurate decisions.

### 3.3. Twin-Delayed Deep Deterministic Policy Gradient

**Figure 4:**
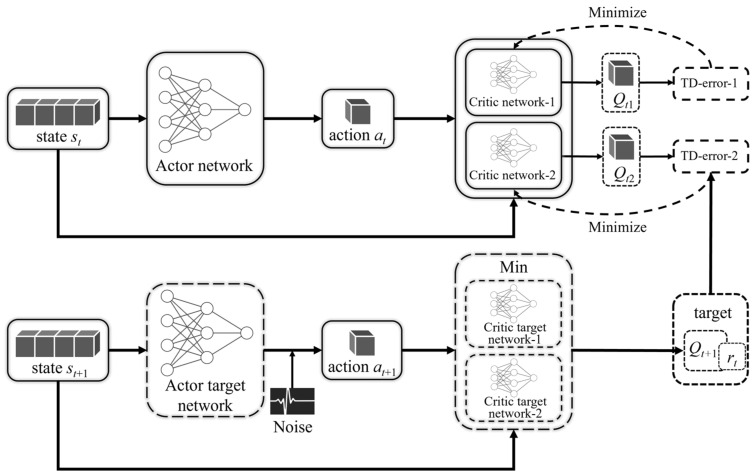
TD3 algorithm improvement structure.

Although the classical DDPG algorithm has achieved satisfactory results in many tasks, the traditional actor–critic framework suffers from the bias and variance problems associated with function approximation, considering the cumulative nature of the DRL iterative process. Specifically, on the one hand, the variance can cause overestimation, while at the same time, high variance can cause the accumulation of errors, which in turn makes the system less stable. Considering the characteristics of time-delayed control systems, high variance means more aggressive control behavior and high deviation means gradual deviation from the steady state, and the agent gains more benefit but also takes more risk, which is more likely to cause instability of the control system. Therefore, we need to suppress both overestimation and cumulative error. Based on the above practical control requirements, this study uses the TD3 algorithm [51] as the basic algorithmic framework, i.e., adding techniques such as clipped double Q-learning, delayed policy updates, and target policy smoothing to the DDPG framework. The framework improvement of the TD3 algorithm is shown in Figure 4.

When building a controller using the TD3 algorithm, first, randomly initialize the critic network $Q_{1}\left(s,a|\theta_{Q1}\right)$, $Q_{2}\left(s,a|\theta_{Q2}\right)$, and actor network $\mu \left(s|\theta_{\mu }\right)$ with parameters $\theta_{Q1}$, $\theta_{Q2}$, and $\theta_{\mu }$, respectively. At the same time, we need to initialize target networks $Q_{1}^{\prime }\left(s,a|\theta_{Q1}^{\prime }\right)$, $Q_{2}^{\prime }\left(s,a|\theta_{Q2}^{\prime }\right)$, and $\mu^{\prime }\left(s|\theta_{{}_{\mu }}^{\prime }\right)$ with parameters $\theta_{{}_{Q1}}^{\prime }$, $\theta_{{}_{Q2}}^{\prime }$, and $\theta_{\mu }^{\prime }$, respectively. Then, synchronize the parameters:

(7) $$\theta_{Q1}\to \theta_{Q1}^{\prime },\theta_{Q2}\to \theta_{Q2}^{\prime },\theta_{\mu }\to \theta_{\mu }^{\prime }$$

In the learning phase, we set the capacity of the experience buffer and the number of episodes. After initializing the state $s_{t}$, the actions are selected based on the current actor network and exploration noise:

(8) $$a_{t}=\mu \left(s_{t}|\theta_{\mu }\right)+\xi_{t}$$

where $\xi_{t}∼N_{t}\left(0,\sigma \right)$.

Performing actions in the environment and obtaining rewards $r_{t}$ and new state $s_{t+1}$. We store these experiences in the form of samples of $\left(s_{t},a_{t},r_{t},s_{t+1}\right)$ that are transformed into the experience buffer. When the experience buffer reaches a certain number of samples, we randomly sample a mini-batch of experience–transfer samples $\left(s_{i},a_{i},r_{i},s_{i+1}\right)$ from it to perform parameter updates. Let

(9) $$y_{i}=r_{i}+\gamma \min_{j=1,2}Q_{j}^{\prime }\left(s_{i+1},\mu^{\prime }\left(s_{i+1}|\theta_{\mu }^{\prime }\right)|\theta_{Qj}^{\prime }+\zeta \right),\zeta ∼clip\left(N\left(0,\tilde{\sigma }\right),-c,c\right)$$

where γ is the discount factor and takes the value of 0.99, which is taken to be equivalent to considering the situation after 100 time steps. *c* is the noise clipping, which in this study is taken as 0.5. The clipped double Q-learning idea and target policy smoothing idea of TD3 are reflected here. With clipped double Q-learning, the value target cannot introduce any additional overestimation bias using the standard Q-learning target. While this update rule may induce an underestimation bias, this is far preferable to overestimation bias, as unlike overestimated actions, the value of underestimated actions will not be explicitly propagated through the policy update [51]. Moreover, the effect of inaccuracy caused by function approximation error can be effectively reduced by target policy smoothing.

Update the parameters $\theta_{Qj}$ of the critic network according to the minimization loss function, i.e.,

(10) $$\nabla_{\theta_{Qj}}L\left(\theta_{Qj}\right)=\frac{1}{N}{\sum_{i}\left(y_{i}-Q_{j}\left(s_{i},a_{i}|\theta_{Qj}\right)\right)}^{2}\nabla_{\theta_{Qj}}Q_{j}\left(s_{i},a_{i}|\theta_{Qj}\right),j=1,2$$

where *N* is the size of the mini-batch sample, whose value we take as 128 in this study. According to our experience, the size of the mini-batch should be related to the complexity of the problem being studied. The parameters $\theta_{\mu }$ of the actor network are updated according to the objective maximization function, i.e.,

(11) $$\nabla_{\theta_{\mu }}J\left(\mu \right)=\frac{1}{N}\sum_{i}\left(\nabla_{a}Q\left(s,a|\theta_{Q}\right)|{}_{s=s_{i},a=\mu \left(s_{i}\right)}\cdot \nabla_{\theta_{\mu }}\mu \left(s|\theta_{\mu }\right)|{}_{s_{i}}\right)$$

Finally, in order to avoid unknown oscillations affecting convergence during gradient descent to ensure that the update of the network can balance stability and rapidity, we perform the soft update process for the network parameters, i.e.,

(12) $$\begin{array}{l} \theta_{Qj}^{\prime }\leftarrow \varepsilon \theta_{Qj}+\left(1-\varepsilon \right)\theta_{Qj}^{\prime },j=1,2\\ \theta_{\mu }^{\prime }\leftarrow \varepsilon \theta_{\mu }+\left(1-\varepsilon \right)\theta_{\mu }^{\prime } \end{array}$$

where ε is the soft update factor, since the delay control system is a high-dimensional unstable system, ε = 0.001 is taken in this study to satisfy the robustness of the policy to some extent.

In addition, the critic network is updated twice as often as the actor network in order to first minimize the errors induced by the value estimation before introducing policy updates. Soft updating of network parameters and delayed policy updating ensure target stabilization and thus reduce error increase. TD3 is summarized in Algorithm 1.
**Algorithm 1 TD3**1Randomly initialize critic networks $Q_{1}\left(s,a|\theta_{Q1}\right)$, $Q_{2}\left(s,a|\theta_{Q2}\right)$, and actor network $\mu \left(s|\theta_{\mu }\right)$ with random parameters $\theta_{Q1}$, $\theta_{Q2}$, and $\theta_{\mu }$.2Initialize target networks $\theta_{Q1}\to \theta_{Q1}^{\prime },\theta_{Q2}\to \theta_{Q2}^{\prime },\theta_{\mu }\to \theta_{\mu }^{\prime }$
3Initialize replay buffer Ω
4**for** episode = 1 to Max episodes, **do**5 Initialize a Gaussian random process $\xi_{t}∼N_{t}\left(0,\sigma \right)$ for action exploration.6 Receive the initial observation state $s_{0}$ of the environment.7 **for**
*t* = 1 to Max steps, **do**8  Select action $a_{t}=\mu \left(s_{t}|\theta_{\mu }\right)+\xi_{t}$ according to the current policy and exploration noise.9  Execute action $a_{t}$ and observe reward $r_{t}$ and observe the new state $s_{t+1}$.10  Store transition tuple $\left(s_{t},a_{t},r_{t},s_{t+1}\right)$ in Ω
11  Sample mini-batch of N transitions $\left(s_{i},a_{i},r_{i},s_{i+1}\right)$ from Ω
12  Set $y_{i}=r_{i}+\gamma \min_{j=1,2}Q_{j}^{\prime }\left(s_{i+1},\mu^{\prime }\left(s_{i+1}|\theta_{\mu }^{\prime }\right)|\theta_{Qj}^{\prime }+\zeta \right),\zeta ∼clip\left(N\left(0,\tilde{\sigma }\right),-c,c\right)$
13  Update parameters $\theta_{Qj}$: $\nabla_{\theta_{Qj}}L\left(\theta_{Qj}\right)=\frac{1}{N}{\sum_{i}\left(y_{i}-Q_{j}\left(s_{i},a_{i}|\theta_{Qj}\right)\right)}^{2}\nabla_{\theta_{Qj}}Q_{j}\left(s_{i},a_{i}|\theta_{Qj}\right),j=1,2$
14  **if**
*t* mod delayed update frequency, **then**15   Update the parameters $\theta_{\mu }$: $\nabla_{\theta_{\mu }}J\left(\mu \right)=\frac{1}{N}\sum_{i}\left(\nabla_{a}Q\left(s,a|\theta_{Q}\right)|{}_{s=s_{i},a=\mu \left(s_{i}\right)}\cdot \nabla_{\theta_{\mu }}\mu \left(s|\theta_{\mu }\right)|{}_{s_{i}}\right)$
16   Soft update target networks: $\begin{array}{l} \theta_{Qj}^{\prime }\leftarrow \varepsilon \theta_{Qj}+\left(1-\varepsilon \right)\theta_{Qj}^{\prime },j=1,2\\ \theta_{\mu }^{\prime }\leftarrow \varepsilon \theta_{\mu }+\left(1-\varepsilon \right)\theta_{\mu }^{\prime } \end{array}$
17  **end if**
18 **end for**
19**end for**

## 4. Controller Model

Solving a new problem using DRL involves the creation of an environment, so in this section, we focus on the environment components involved in the controller model, namely, the state, action, reward, and transfer function. Among them, the transfer function of DRL is the dynamics model introduced in Section 2, which will not be repeated here.

### 4.1. State

The state is the information that describes the environment, and the RL environment must provide the algorithm with enough information to solve the problem. In the active suspension delay control system, multidimensional information such as displacement, velocity, acceleration, control force, and time are included. These high-level states are the raw states. The raw state should contain all the information relevant to the problem, but it is often difficult to learn because it usually has a lot of redundancy and background noise. Raw and complete information provides more freedom, but extracting and interpreting useful signals from it is a much heavier burden. Therefore, the selection and design of the state space becomes particularly important.

Based on the important suspension performance parameters included in the dynamics model, the following state variables are chosen to characterize the state space considering the control performance requirements of the active suspension. The designed state space takes into account both real-world constraints and observability cases under the influence of sensor arrangements.

(13) $$s={\left[\begin{array}{cccc} {\ddot{x}}_{b} & {\dot{x}}_{b} & {\dot{x}}_{b}-{\dot{x}}_{u} & x_{b}-x_{u} \end{array}\right]}^{T}$$

The state information takes into account the ride comfort, actuator efficiency, and suspension travel of the suspension system. This state contains much less information than the original state, but it contains more straightforward information for the algorithm and is easier to learn.

Further, the agent further preprocesses the designed states for its own use. In order to improve the generalization performance of the controller model, this study normalizes the states, and the final state vector is represented as

(14) $$s={\left[\begin{array}{cccc} \frac{{\ddot{x}}_{b}}{\lambda_{1}} & \frac{{\dot{x}}_{b}}{\lambda_{2}} & \frac{{\dot{x}}_{b}-{\dot{x}}_{u}}{\lambda_{3}} & \frac{x_{b}-x_{u}}{\lambda_{4}} \end{array}\right]}^{T}$$

where λ is the normalized coefficient of each state variable.

### 4.2. Action

Action is the output of the agent, which is the amount of control output by the controller. Actions change the environment by transitioning the dynamics model to the next state and into the next round of iterations. How the action is designed affects the ease of control of the system and thus directly affects the problem’s difficulty. In this study, the action is the control force of the active suspension actuator, i.e.,

(15) $$F_{t}=a_{t}=\mu \left(s_{t}|\theta_{\mu }\right)$$

where $\mu \left(s|\theta_{\mu }\right)$ is the actor network. It should be noted that due to the delay, the control force in the actual system often appears as

(16) $$a_{t+\tau +\tau_{a}}=\mu \left(s_{t}|\theta_{\mu }\right)$$

Considering the specific performance constraints of the actuator, adding the double truncation constraint to the actor network, the final action is represented as

(17) $$a=clip\left(a_{t+\tau +\tau_{a}},F_{\min},F_{\max}\right)$$

In this study, based on the saturation constraint of the actuator, we set $F_{\min}=-3\;\mathrm{kN}$ and $F_{\max}=3\;\mathrm{kN}$.

### 4.3. Reward

The reward signal is used to define the objective that the agent should maximize to guide the agent’s exploration to obtain a better policy from a global level. The reward function can be somewhat analogous to the objective function of a traditional control problem, so reward design is an important issue in DRL. In the design of an active suspension controller considering delay, the control performance requirements of multiple objectives need to be considered.

The first issue is the ride comfort, which is closely related to the vertical acceleration of the vehicle body. Therefore, the impact of road unevenness on the body is avoided, and the vertical acceleration is reduced while a suitable active control force is needed.

Secondly, the practical constraints of suspension travel need to be considered. Dynamic suspension travel needs to satisfy the following inequalities within a safe range:

(18) $$\left|x_{b}\left(t\right)-x_{u}\left(t\right)\right|\leq f_{d}$$

where $f_{d}$ is the maximum travel value of the suspension, and in this study, let $f_{d}=0.15\;m$. The value is determined by referring to the literature [52] and considering the actual constraints of the suspension obtained.

Then, the grounding of the suspension needs to be considered, i.e., the following inequalities need to be satisfied to ensure the handling stability of the vehicle:

(19) $$k_{u}\left|x_{u}\left(t\right)-w\left(t\right)\right|\leq F_{m}$$

where $F_{m}$ is the static load of the tire, and its calculation formula is

(20) $$F_{m}=\left(m_{b}+m_{u}\right)g$$

Finally, the control characteristics of the actuator need to be considered. The actuator delay $\tau_{a}$ has a close relationship with the total delay of the whole system, so in order to suppress the effect of delay at the physical level, we should ensure that the control force is relatively stable in a relatively small interval as much as possible.

In summary, the reward function is defined as

(21) $$r=-\left(k_{1}{\left|{\ddot{x}}_{b}\right|}^{2}+k_{2}{\left|x_{b}-x_{u}\right|}^{2}+k_{3}{\left|x_{u}-w\right|}^{2}+k_{4}{\left|F\right|}^{2}\right)$$

where $k_{1}=0.7,k_{2}=0.1,k_{3}=0.1,k_{4}=0.1$ are the weight coefficients of the balanced multi-objective optimization problem.

It should be noted that the agent’s reward function in the training phase references the state information of the system after the control force has been applied after the delay. In contrast, the state referenced by the actor network and critic network is the current state. In other words, the delayed control system in the experimental phase is equivalent to an open-loop control system.

## 5. Simulation and Results

This section describes the simulation and results used to verify the proposed control algorithm in the simulation environment built by MATLAB2023a/Simulink. First, the specific environment and agent-related network information required for the simulation experiments were introduced and set up. Then, parallel experiments were conducted for 10 ms, 20 ms, and 30 ms defined delay conditions to demonstrate the control performance of the proposed algorithm for deterministic delay control. In addition, we established a semi-regular delay control condition based on the fuzzy relationship between actuator control force and delay to test the excellent control performance of the proposed algorithm. Finally, we established a severe operating condition with uncertain delay to test the anti-disturbance performance of the proposed algorithm and its improvement of ride comfort.

### 5.1. Implementation Details

The active suspension system dynamics model with consideration of delay introduced in Section 2 was used to build the environment required for training the DRL, and the model parameters and values are listed in Table 1. The road information used for training the agent is shown in Figure 2, and a deterministic delay of 30 ms was added to the network during training.

**Figure 5:**
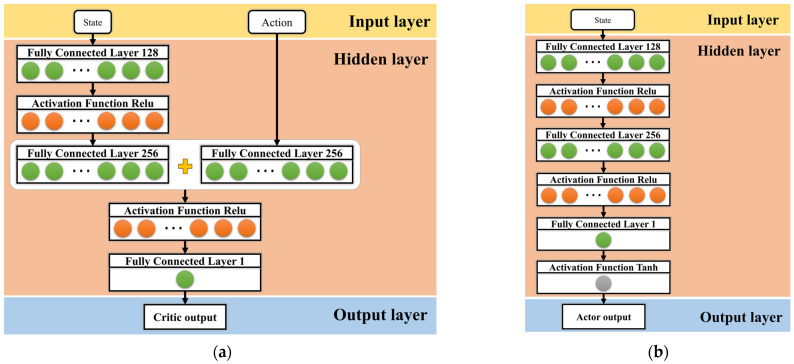
Critic and actor network. (**a**) Network architecture created for the critic, and (**b**) network architecture created for the actor. It should be noted that the last fully connected layer in the critic network directly outputs the result without the need for activation.

The critic and actor network used for the agent were specifically designed for the control of active suspension systems, as shown in Figure 5, and the hyperparameters used to train the network are shown in Table 2. In order to better verify the performance of the proposed algorithm in this study, we chose the active suspension DDPG control architecture proposed in the literature [53] as a baseline for comparison. The hyperparameters of the baseline algorithm were used in this study, and additional hyperparameters were selected based on the original TD3 algorithm. In addition, we performed combinatorial experiments on some of the hyperparameters; see Appendix B.

### 5.2. Deterministic Delayed Conditions

Due to the random nature of the road excitation during the actual driving of the vehicle, we selected a random class C road as the random excitation. The proposed active control algorithm of the delay system was used to study the dynamics of the suspension system under random excitation. One study [20] showed that the classical control method could not prevent unstable divergence in an active suspension system with a 24 ms time delay. Based on this conclusion, it was determined that the delay times were designed to be similar to 10 ms, 20 ms, and 30 ms.

**Figure 6:**
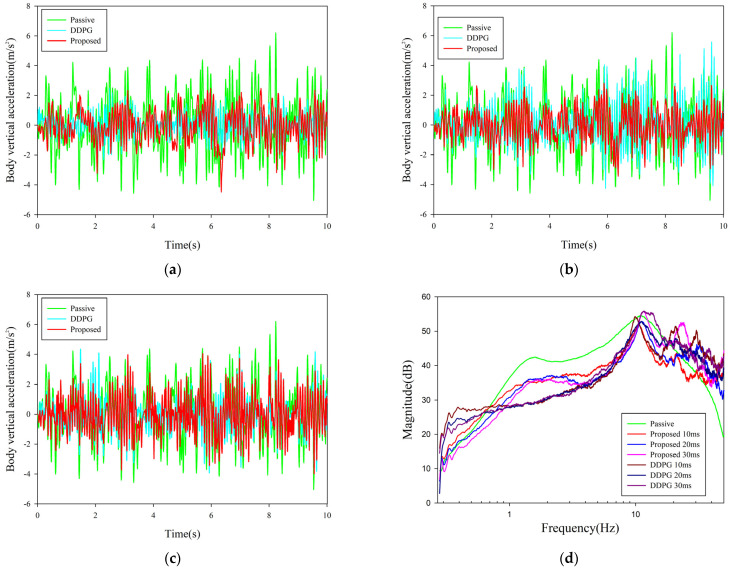
Control performance with deterministic time delay. (**a**–**c**) Vehicle body acceleration curves under 10 ms, 20 ms, and 30 ms delay, respectively. (**d**) Frequency response of ${\ddot{x}}_{b}/w$.

We quantitatively evaluated the intensity of body vibration under DRL control using root mean square (RMS) values of acceleration. In addition, we also analyzed the frequency response of the body acceleration to a random road. The RMS results of the proposed algorithm with different time delays in the simulation environment were compared with passive results, as tabulated in Table 3. The simulation comparison results of the body acceleration and the frequency response are shown in Figure 6. In addition, we compared the proposed algorithm with the most classical and effective DDPG algorithm [53]; the comparison results are also presented in the graphs.

As we can see from the graphs, the proposed control algorithm optimized the ride comfort by 43.58%, 44.9%, and 32.28% for 10 ms, 20 ms, and 30 ms deterministic delays, respectively, compared to the passive suspension. Although the control algorithm of the proposed algorithm is slightly inferior to that of DDPG under the low latency condition of 10 ms, the control performance of the proposed algorithm improves by 25.56% compared to that of DDPG under the latency condition of 20 ms. Further, under the large delay condition of 30 ms, the proposed algorithm still maintains the optimization result of 32.28% compared to DDPG, which cannot maintain stability and crashes. The above results clearly demonstrate the superior control performance of the proposed algorithm. Although the proposed algorithm exhibited good control performance at deterministic delays, realistic control systems did not always have deterministic delays. Deterministic delay is equivalent to adding a deterministic dimension to the overall control system, which is still solvable to some extent. Therefore, the study of delayed control systems requires more in-depth discussion and analysis.

### 5.3. Semi-Regular Delayed Conditions

The amount of delay in a control system is often closely related to the actual actuation capability of the actuator, so some scholars used an integrated system model that includes the actuator dynamics to design the controller. To simulate this characteristic, we developed a semi-regular delay condition, specifically using the following rules:

(22) $$\tau_{a}=\{\begin{array}{c} \delta ,0<\left|\Delta F\right|\leq f\\ 2\delta ,f<\left|\Delta F\right|\leq 2f\\ 3\delta ,2f<\left|\Delta F\right|\leq 3f \end{array}$$

where δ is the unit delay amount, and its value was taken as 10 ms in this study. f is the unit amount determined according to the maximum limiting control force of the actuator, and its value was taken as 2 kN in this study. This value is chosen by considering the bandwidth of the active suspension actuator and can actually be obtained by testing the actuation force response characteristics of the active suspension actuator under different loads.

**Figure 7:**
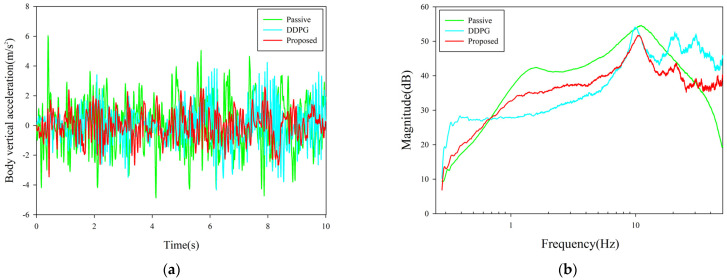
Control performance with semi-regular time delay. (**a**) Vehicle body acceleration curve under semi-regular time delay. (**b**) Frequency response of ${\ddot{x}}_{b}/w$.

The semi-regular delay condition was based on the fuzzy relationship between actuator delay and actuation capacity, and the delay was graded in steps. It can make the system simple while retaining the actuator’s role to a greater extent, so this condition had stronger practical significance for testing the control algorithm. The body acceleration and frequency response with the semi-regular delay condition are shown in Figure 7.

In the semi-regular delay condition, the RMS result of the proposed algorithm is 0.9529 m/s^2^, and the ride comfort is optimized by 44.13%. In comparison, the RMS value under the DDPG baseline control is 1.1321 m/s^2^, while the control performance of the proposed algorithm exceeds the DDPG baseline by 15.8%. We can see that the proposed algorithm still maintained good control performance in the operating conditions where the fuzzy characteristics of the actuator were considered.

### 5.4. Uncertain Delay Conditions

The previous consideration of deterministic delay and semi-regular delay conditions amounted to adding one or more dimensions of uncertainty to the delay control system. Further, the delay in a practical time-delay control system was often uncertain. Mathematically and theoretically, this environment was closer to an infinite-dimensional control system. Therefore, we set up simulation experiments under uncertain delay time working conditions to simulate a more severe environment. Under uncertain delay conditions, DDPG fails to converge due to its inability to adapt to such a high-dimensional working condition.

Specifically, the delay time was set to satisfy the uniform distribution of the following equation:

(23) $$\tau_{a}=\nu ,\nu ∼U\left(10,40\right)$$

**Figure 8:**
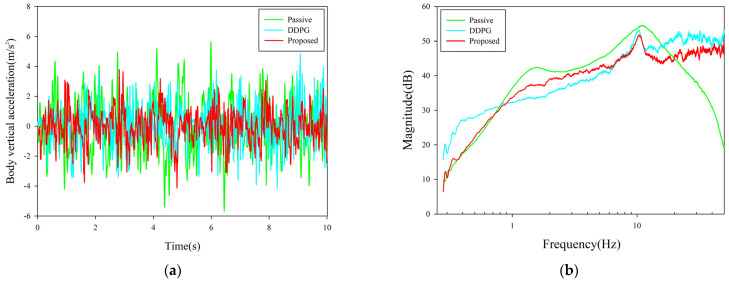
Control performance with uncertain time delay. (**a**) Vehicle body acceleration curve under uncertain time delay. (**b**) Frequency response of ${\ddot{x}}_{b}/w$.

The body acceleration and frequency response under the uncertain delay conditions are shown in Figure 8. The value of RMS under the control of the proposed algorithm was 1.1116 m/s^2^ in the uncertain delay condition, and the optimization of ride comfort reached 37.56%. In comparison, the RMS value under the DDPG baseline control is 1.2528 m/s^2^, while the control performance of the proposed algorithm exceeds the DDPG baseline by 11.3%. It can be seen that the control performance of the proposed algorithm only showed a small degradation in the case of a sharp increase in system complexity. In Figure 8b, the response of the proposed algorithm to road vibration shows a slight increase in a certain range in the high-frequency part. This is due to the fact that the uncertain delay condition is an extremely severe condition, so the DRL algorithm will prioritize the control needs in the low-frequency section. A more in-depth study will be carried out for high-frequency control.

It should be noted that in the comparative experiments, the DDPG algorithm was unable to complete the full verification due to triggering the termination condition within the episode described in Equation (18) under the conditions of a 30 ms large delay, a semi-regular delay, and an uncertain delay. In order to conduct the comparative experiments, we had to remove the relevant termination conditions. In comparison, the proposed algorithm consistently did not trigger the termination condition, ensuring the safety of the driving process. Furthermore, in order to verify the generalization performance of the proposed algorithm in different environments, we conducted experiments by varying the speed. The control results are shown in Table 4. The table indicates that the proposed algorithm shows good control performance at different speeds.

## 6. Discussion

In this study, we made some beneficial attempts using DRL to solve the challenging problem of time delay control in active suspension systems. We set deterministic, semi-regular, and uncertain time delays to simulate the changes from ideal working conditions to real working conditions and then to harsh working conditions, thereby testing the control performance of the proposed algorithm. Under deterministic delay, the proposed algorithm demonstrated good control performance at working conditions of 10 ms, 20 ms, and 30 ms, surpassing the DDPG baseline and maintaining good stability even under larger time delays. In addition, the proposed algorithm effectively suppressed the first resonance peak of road excitation and body, and improved ride comfort. The proposed algorithm includes predictions of future rewards, thus possessing stronger robustness to a certain extent. This condition corresponds to a relatively ideal working environment for the actuator, where stable fixed delay is desirable. However, under actual conditions, system delay is often closely related to the actuator’s manufacturing capability and response. Therefore, we designed semi-regular delay conditions to simply simulate this characteristic. The simulation results also reflected the good control performance of the proposed algorithm and its improvement in ride comfort. We believe that these results are due to the fact that the proposed algorithm, based on predicting the future, has imposed planning on the output of control force, keeping it within a small range of variation that better aligns with the actuator’s response characteristics. Furthermore, uncertain conditions are relatively harsh working conditions, and it is necessary to conduct simulations under such conditions to better test the performance of the proposed algorithm. It can be seen that under such conditions, the proposed algorithm can still maintain a 37.56% optimization effect. We believe this is because, for the infinite-dimensional delay control system, the data-driven algorithm bypasses the analysis at the high-dimensional level and directly performs end-to-end analysis. To a certain extent, it corresponds to re-architecting a solver that simulates a high-dimensional environment, and this approach is undoubtedly novel and effective. Of course, further research is needed to verify its effectiveness. It is encouraging that Baek et al. [54] and Li et al. [55] have attempted to apply DRL to robotics, Zhu et al. [56] have applied it in Mobile Sensor Networks, and Chen et al. [57] have generalized it even more to continuous control. At the same time, it is also important for us to focus on choosing more suitable algorithms for the control of delay systems. In recent years, PPO [58] has been widely applied in the industry due to its robustness and performance. The characteristics of PPO in parameter tuning and policy updating provide us with new ideas for our future research, which will be a valuable direction for future studies.

Furthermore, the generalization ability of the algorithm has always been a controversial issue for learning-based algorithms. For this reason, we conducted simulations at different speeds, and the results showed that the proposed algorithm maintained over 30% comfort optimization from 10 m/s to 50 m/s, which covers almost all driving speeds in reality. Moreover, to apply the proposed algorithm in the real world, the complexity of the algorithm must be considered, and its real-time calculation performance must be examined. The trained DRL controller has 33,537 parameters, and it only takes 5.6 ms to compute on a typical PC (Intel Core i9-12900KF, 16 GB RAM). Therefore, the proposed controller will not be a problem in terms of real-time implementation.

## 7. Conclusions

This paper proposed an active suspension DRL control algorithm considering time delay to study the uncertain delay problem in the actual control system of active suspension. Firstly, a dynamics model of the active suspension system considering time delay was established. Secondly, the TD3 algorithm was enhanced by incorporating delay, enabling the agent to explore more robust policies. Finally, simulation experiments were conducted under three different experimental conditions: deterministic delay, semi-regular delay, and uncertain delay. The proposed algorithm’s control performance was evaluated, and experimental validation was performed at various speeds. The results illustrate the algorithm’s effectiveness in mitigating the impact of uncertain delay on the active suspension system, resulting in significant improvements in ride comfort optimization. Specifically, the proposed algorithm achieved comfort optimization rates of 43.58%, 44.9%, and 32.28% for deterministic delays of 10 ms, 20 ms, and 30 ms, respectively. Additionally, it obtained optimization rates of 44.13% and 37.56% for semi-regular and uncertain delay conditions, respectively. Furthermore, when compared to the DDPG baseline algorithm, the proposed algorithm demonstrates excellent stability and convergence even under complex delay conditions.

Despite satisfactory results in the current research, the important characteristic of time delay still requires further investigation. In future work, we aim to enhance our understanding of the relationship between delay and control performance by incorporating an integrated system model that accounts for actuator dynamics into the DRL environment. By relying on a comprehensive model environment, the agent can derive improved control policies that are better suited for real-world vehicle deployment scenarios.

## Figures and Tables

**Table 1:** Environment model parameters.

Parameters	Value	Parameters	Value
$m_{b}$	400	$m_{u}$	40
$k_{b}$	20,000	$k_{u}$	200,000
v	20	τ	0
$S_{q}$	256	$c_{u}$	0
$c_{b}\left(\mathrm{active}\right)$	0	$c_{b}\left(\mathrm{passive}\right)$	1500

**Table 2:** Agent hyperparameter.

	Hyperparameter	
	Item	Value
Critic	Learning rate	1 × 10^−3^
	Gradient threshold	1
	L2 Regularization factor	1 × 10^−4^
Actor	Learning rate	1 × 10^−2^
	Gradient threshold	1
Agent	Sample time	0.01
	Target smoothing factor	1 × 10^−3^
	Experience buffer length	1 × 10^6^
	Discount factor	0.99
	Mini-batch size	128
	Soft update factor	1 × 10^−3^
	Delayed update frequency	2
	Noise clipping	0.5
	Noise variance	0.6
	The decay rate of noise variance	1 × 10^−5^
Training process	Max episodes	2000
	Max steps	1000

**Table 3:** RMS values of body acceleration in deterministic delayed conditions.

Controller	Passive	10 ms		20 ms		30 ms	
		Proposed	DDPG	Proposed	DDPG	Proposed	DDPG
$\begin{array}{c} {\ddot{x}}_{b}\\ \left(m/s^{2}\right) \end{array}$	1.8778	1.0595 (+43.58%)	0.7772 (+58.61%)	1.0347 (+44.9%)	1.3901 (+25.97%)	1.2716 (+32.28%)	1.5439 (+17.78%)

**Table 4:** RMS values of body acceleration at different speeds.

Speed (m/s)	Passive	Proposed	Optimization
10	1.265	0.8853	30.02%
15	1.5461	0.9885	36.06%
20	1.7803	1.1116	37.56%
25	1.9837	1.2049	39.26%
30	2.1645	1.2794	40.89%
35	2.3275	1.353	41.87%
40	2.476	1.4122	42.96%
45	2.6123	1.4705	43.71%
50	2.7383	1.5175	44.58%

## Data Availability

Not applicable.

## Appendix A. Notations and Abbreviations

**Table A1:** The notations used in the paper.

Parameters	Notation	Unit
τ	Inherent delay	s
$\tau_{a}$	Actuator delay	s
$m_{b}$	Sprung mass	kg
$m_{u}$	Unsprung mass	kg
$k_{b}$	Spring stiffness	N/m
$k_{u}$	Equivalent tire stiffness	N/m
$c_{b}$	Equivalent damping factor	N⋅s/m
$x_{b}$	Vertical displacement of the sprung mass	m
$x_{u}$	Vertical displacement of the unsprung mass	m
w	Road displacement	m
$F=u\left(t-\tau -\tau_{a}\right)$	Active suspension actuator control force	N
v	Speed	m/s
$f_{0}$	Lower cutoff frequency	—
$S_{q}\left(n_{0}\right)$	Road unevenness coefficient	—
$w_{0}$	Uniformly distributed white noise	—
$n_{00}$	Spatial cutoff frequency	—
$s_{t}$	State at time *t*	—
$a_{t}$	Action at time *t*	—
$s_{t+1}$	State at time *t* + 1	—
$r_{t}$	Reward at time *t*	—
$Q\left(s,a|\theta_{Q}\right)$	Critic network	—
$\mu \left(s|\theta_{\mu }\right)$	Actor network	—
$Q_{}^{\prime }\left(s,a|\theta_{Q}^{\prime }\right)$	Target critic network	—
$\mu^{\prime }\left(s|\theta_{{}_{\mu }}^{\prime }\right)$	Target actor network	—
γ	Discount factor	—
ε	Soft update factor	—
$\xi_{t}$	Exploration noise	—
ζ	Smoothing noise	—
λ	Normalized coefficient	—
$f_{d}$	Maximum travel value of the suspension	—
$F_{m}$	Static load of the tire	—
k	Weight coefficient	—
δ	Unit delay amount	s
f	Unit amount determined force	kN

**Table A2:** The list of abbreviations.

Abbreviation	Full Name
DDPG	Deep Deterministic Policy Gradient
DL	Deep Learning
DQN	Deep Q Network
DRL	Deep Reinforcement Learning
LQR	Linear Quadratic Regulator
MDP	Markov Decision Processes
PPO	Proximal Policy Optimization
RL	Reinforcement Learning
RMS	Root Mean Square
SAC	Soft Actor–Critic
TD3	Twin-Delayed Deep Deterministic Policy Gradient

## Appendix B. Hyperparametric Portfolio Experiment

In this study, a specifically designed deep neural network architecture was chosen to reduce computational complexity to meet the requirements of the controller in the real world. In order to address the exploration and utilization challenges, the learning rate of actor and critic networks is also an important influence. Therefore, we have investigated the hyperparameter combinations of these two components separately, which are shown in Table A3 and Table A4, respectively.

**Table A3:** Number of neurons in the deep neural network.

Neuronal Assemblies	32–64	64–128	128–256	256–512
Optimization	24.99%	30.30%	**35.62%**	32.82%

**Table A4:** Critic network and actor network learning rate. The first behavioral critic network learning rate and the first column is the actor network learning rate.

Optimization	0.1	0.01	0.001
0.1	16.77%	23.79%	23.72%
0.01	28.59%	31.98%	28.82%
0.001	31.00%	**35.62%**	32.81%
